# The pattern from the first three rounds of vaccination: declining vaccination rates

**DOI:** 10.3389/fpubh.2023.1124548

**Published:** 2023-05-12

**Authors:** Jian Wu, Xinghong Guo, Xue Zhou, Meiyun Wang, Jianqin Gu, Yudong Miao, Clifford Silver Tarimo, Yilin He, Yuhan Xing, Beizhu Ye

**Affiliations:** ^1^Department of Health Management, College of Public Health, Zhengzhou University, Henan, China; ^2^Henan Province Enginering, Research Center of Health Economy and Health Technology Assessment, Zhengzhou, Henan, China; ^3^Department of Public Utilities Management, College of Health Management, Mudanjiang Medical University, Mudanjiang, Heilongjiang, China; ^4^Henan Provincial People’s Hospital, People’s Hospital of Zhengzhou University, Zhengzhou, Henan, China; ^5^School of Medicine, Southern University of Science and Technology, Shenzhen, Guangdong, China

**Keywords:** COVID–19, vaccination rates, booster vaccination, chronic disease, China

## Abstract

**Introduction:**

Vaccination rates for the COVID-19 vaccine have recently been stagnant worldwide. We aim to analyze the potential patterns of vaccination development from the first three doses to reveal the possible trends of the next round of vaccination and further explore the factors influencing vaccination in the selected populations.

**Methods:**

On July 2022, a stratified multistage random sampling method in the survey was conducted to select 6,781 people from 4 provinces China, who were above the age of 18 years. Participants were divided into two groups based on whether they had a chronic disease. The data were run through Cochran-Armitage trend test and multivariable regression analyses.

**Results:**

A total of 957 participants with chronic disease and 5,454 participants without chronic disease were included in this survey. Vaccination rates for the first, second and booster doses in chronic disease population were93.70% (95% CI: 92.19–95.27%), 91.12% (95%CI: 94.43–95.59%), and 83.18% (95%CI: 80.80–85.55%) respectively. By contrast, the first, second and booster vaccination rates for the general population were 98.02% (95% CI: 97.65–98.39%), 95.01% (95% CI: 94.43–95.59%) and 85.06% (95% CI: 84.11–86.00%) respectively. The widening gap in vaccination rates was observed as the number of vaccinations increases. Higher self-efficacy was a significant factor in promoting vaccination, which has been observed in all doses of vaccines. Higher education level, middle level physical activity and higher public prevention measures play a positive role in vaccination among the general population, while alcohol consumption acts as a significant positive factor in the chronic disease population (*p* < 0.05).

**Conclusion:**

As the number of vaccinations increases, the trend of decreasing vaccination rate is becoming more pronounced. In future regular vaccinations, we may face low vaccination rates as the increasing number of infections and the fatigue associated with the prolonged outbreak hamper vaccination. Measures need to be found to counter this downward trend such as improving the self-efficacy of the population.

## Introduction

In order to minimize the impact of the COIVD-19 pandemic on people’s work and life order as well as economic and social development, the Chinese government has issued a new epidemic prevention policy, which lists measures 10 to further achieve precise epidemic prevention and control (1). The policy eases previously strict quarantine measures and increases population mobility to rejuvenate society, which could lead to an increase in the speed of the virus spreading among people. Establishing herd immunity is an efficient method for preventing the spread of COVID-19, and vaccination is one of the best cost-effective method for achieving herd immunity (2), curb the spread of COVID-19 and mitigate the consequence of infections (3–5). However, due to the decline of immunity and the virus mutation, primary vaccination shows low protective effect (6, 7). Booster vaccines were hence introduced to strengthen the efforts to fight the disease and prevent severe epidemic. The vaccination rate is crucial to establish herd immunity, and the higher the vaccination rate, the better the vaccine’s protective effect on the population (8). China currently has a widespread third dose of the vaccine, while the fourth dose has yet to be widely rolled out. Increase in both primary and booster vaccination rates appear to have stalled according to the data on changes in vaccination rates over time in China (9). How to boost the seemingly stalled vaccination rates and prepare for the next round of vaccination to strengthen herd immunity is an urgent topic in the current context of COVID-19.

People with different characteristics may have different opinions and behaviors about the same thing. For example, people with chronic diseases show different attitudes toward vaccination with the COVID-19 vaccine than the general population. Despite the higher risk of infection and poorer outcomes for infection (10–13), previous research demonstrated that people with chronic diseases are less likely to be vaccinated than the general population (14–16). In China the chronic disease population accounts for 34.3% of the population, which is a significant proportion of the population (17). These two groups may exhibit distinct towards COVID-19 vaccination attitude, and hence if they will not be treated separately in the analysis, some information may be lost. By distinguishing the general population by the presence or absence of chronic diseases and thus identifying unique or common behavioral characteristics and influencing factors of different populations for vaccination with the COVID-19 vaccine, interventions can be implemented with even greater precision and intent to increase vaccination rates.

Currently, most studies revolve around the willingness to vaccinate, and relatively few studies have been conducted on vaccination. There is a significant gap between vaccination willingness and vaccination. The purpose of our study was to analyze the potential patterns of vaccination development from the first three doses to reveal the possible trends of the next round of vaccination and further explore the factors influencing vaccination in the selected populations.

## Method

### Participants and procedures

**Figure 1:**
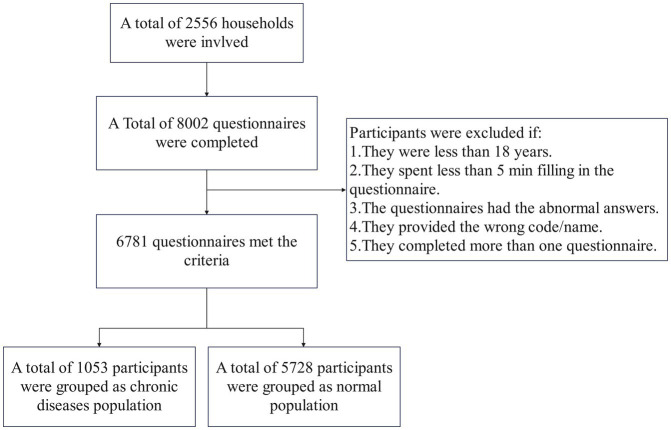
Flowchart of participants selection.

A multicenter and observational household tracking survey was conducted to explore the Dynamic Evolution of the COVID-19 Vaccination Study (DECVS) through a stratified random sample in China. First, we selected four representative provinces from the eastern, central, western and northeastern regions of China, respectively. We then selected one city from each of the selected provinces randomly. Changzhou, Zhongmou, Xi’ning and Mudanjiang represent East, Central, West and Northeast parts of country, respectively. At least two rural areas and two urban areas were chosen from the selected cities, respectively. Finally, families were also selected randomly using an online platform that provides designing and collecting questionnaires services. All members of selected family were involved this survey (age ≥ 18 years) and completed the online or offline questionnaire with the assistance of investigators. If participants did not have access to the Internet or mobile devices, they would be provided with a paper questionnaire. On July 2, 2022, the first stage of DECVS was completed. The participant selection process is shown in Figure 1. This study was deemed exempt from assessment by the Zhengzhou University Life Science Ethics Review Committee’s ethical review board.

### Sample size estimation

The special formula for sample size calculation of cohort study and NCS-PASS 11 software were used to estimate sample size.


$$n=\frac{{\left(Z_{\alpha }\sqrt{2\bar{pq}}+Z_{\beta }\sqrt{p_{0}q_{0}+p_{1}q_{1}}\right)}^{2}}{{\left(p_{1}-p_{0}\right)}^{2}}$$

Assuming significance *a* = 0.05(bilateral) degree of assurance (1-B) = 0.90, according to the results of previous studies, the intention rate of intensive immunization with COVID-19 vaccine in China is 91.61%, so p0-8.39%, and relative risk RR = 0.5 in the exposed group compared with the non-exposed group. If the ratio of the exposed group to the non-exposed group is 1:1, the sample size should be 2050. Taking into account the loss of follow-up and extending the calculated sample size by 20%, the total sample size required is 2,562.

### Patient and public involvement

Patients or the public were not involved in the design, or conduct, or reporting, or dissemination plans of our research.

### Assessment

We conducted a preliminary experiment and obtained 129 data sets. Through the analysis of the pre-survey data, the Cronbach’s Alpha, CFI, TLI and RMSEA of self-efficacy were 0.941, 0.999, 0.998 and 0.038, indicating that the reliability and validity of the questionnaire were good.

Participants with the chronic disease were identified by the question “Do you currently have a chronic medical condition diagnosed by a physician or hospital?” The item had two options: “yes” and “no.” Participants who chose yes were identified as “chronic diseases population” and those who chose no were considered as the “general population.” Participants who answered that they had a chronic condition were asked three further questions: 1) whether they had doctor-confirmed hypertension, 2) Whether you have doctor-confirmed diabetes, and 3) whether you have any other chronic diseases. Participants with chronic diseases were divided into three categories based on the previous three questions: participants with high blood pressure, participants with diabetes, and participants with other chronic diseases. Hypertension is defined as a systolic blood pressure of 140 or greater and a diastolic blood pressure of 90 or greater. Three consecutive measurements with different sphygmomanometers at different times all exceeded the normal value. Diabetes was defined as having symptoms of diabetes and fasting blood glucose greater than or equal to 7 mmol/L, or postprandial blood glucose greater than or equal to 11.1 mmol/L and random blood glucose greater than 11.1 mmol/L. Diabetes mellitus is diagnosed if the patient has no symptoms of diabetes and the fasting blood glucose exceeds 7 mmol/L for both times, or the postprandial blood glucose exceeds 11 mmol/L for two times, or the fasting blood glucose exceeds 7 mmol/L and the postprandial blood glucose exceeds 11 mmol/L.

We designed two items to assess the vaccination status. The items comprised of questions “Have you been vaccinated against COVID-19?” for identifying whether participants have received the first or second dose of vaccine and “Have you completed the booster shot of COVID-19 vaccine?” for identifying whether participants have received the booster vaccines. The item “Have you been vaccinated against COVID-19” has three options, including (A) Yes, I have received and completed fully vaccination, (B) Yes, I have received but not completed fully vaccination, and (C) No, I have not received at all. The item “Have you completed the booster shot of COVID-19 vaccine” had two options, including (1) Yes, (2) No. Participants who chose (C) were considered unvaccinated, those who chose (B) were considered to have received only the first dose of vaccine, those who chose (A) were considered to have completed the full dose of vaccination, and those who chose (A) and (1) were considered to have received the booster dose.

Self-efficacy was measured by 4 questions: 1) Even if it takes time for work or study, I will get a booster shot, 2) Even if no one around me gets it, I will get a booster shot, 3) Even if I have a fear of needles, I will get it and 4) Even if I still need to take protective measures after I get the vaccine, I will get it. Participants can choose one of the five answers (Strongly disagree, disagree, uncertain, agree, and Strongly agree). We assign the answers from 1 to 5 and add up the scores of the 4 questions. Higher scores mean higher self-efficacy. The score of self-efficacy was divided in trichotomies. The scales’ internal consistency reliability was determined with Cronbach’s alpha and demonstrated good reliability of *α* = 0.969.

### Statistical analysis

The Chi-square test was used to compare the characteristics of vaccinated and unvaccinated individuals in response to three doses of vaccine and to determine whether there is a difference in vaccination rates between the general population and those with chronic diseases. Cochran-Armitage trend test was conducted for verifying that if there is a linear trend in vaccination rates in different dimensions. The Bonferroni test was performed for *post Hoc* Multiple Comparisons. A multivariable logistic regression model was conducted for unvaccinated behavior of three doses of vaccine adjusting, respectively, factors. The method used for regression is Enter. All data analyses were conducted by IBM Statistical Package for the Social Sciences (SPSS) 26.0.

## Result

### Demographic characteristics

Of the 6,411 participants included, 957 had chronic disease and 5,454 did not. The demographic characteristics of the participants in both groups are summarized in Table 1. The age of participants with chronic diseases was 59 (50–67) years, while the age of general participants was 41 (33–53) years, indicating that participants with chronic diseases were predominantly elderly, while general participants were predominantly middle-aged. Among participants with chronic diseases, the male–female ratio was 1.2:1, compared to 1:1.2 in the general population. Most of the participants were married, with a low level of education and the majority of people took high-level public health prevention measures and moderate self-efficacy accounted for about half of the participants.

**Table 1:** The demographic characteristic of chronic disease population and general population received the COVID-19 vaccines among China.

Variables	Total *n* (%)		First dose vaccinated (%)		Second dose vaccinated (%)		Booster dose vaccinated (%)	
	Chronic disease population	General population	Chronic disease population	General population	Chronic disease population	General population	Chronic disease population	General population
All participants	957 (14.93)	5,454 (85.07)	93.73(92.19–95.27)	98.02(97.65–98.39)	91.12(89.31–92.92)	95.01(94.43–95.59)	83.18(80.80–85.55)	85.06(84.11–86.00)
Age, years	59 (50–67)^b^	41 (33–53)^b^	–	–	–	–	–	–
Sex			*p* = 0.518	*p* = 0.343	*p* = 0.177	*p* = 0.943	*p* = 0.226	*p* = 0.662
Male	517 (54.02)^a^	2,518 (46.17)^a^	94.20(92.18–96.22)	98.21(97.7–98.73)	92.26(89.95–94.57)	95.04(94.19–95.88)	84.53(81.4–87.65)	84.83(83.43–86.23)
Female	440 (45.98)^a^	2,936 (53.83)^a^	93.18 (90.82–95.55)	97.85 (97.33–98.38)	89.77 (86.93–92.62)	94.99 (94.2–95.78)	81.59 (77.96–85.23)	85.25 (83.97–86.54)
Marital status			*p* = 0.752	*p* = 0.056	*p* = 0.955	*p* = 0.290	*p* = 0.403	*p* = 0.197
Married	993 (94.25)^a^	4,729 (86.34)^a^	93.79 (92.21–95.37)	97.88 (97.46–98.29)	91.13 (89.27–92.99)	95.14 (94.52–95.75)	82.85 (80.42–85.28)	85.3 (84.29–86.32)
Others	60 (5.75)^a^	745 (13.66)^a^	92.73 (85.64–99.81)	98.93 (98.18–99.67)	90.91 (83.07–98.75)	94.23 (92.55–95.91)	93.33 (83.86–102.81)	83.49 (80.82–86.16)
Educational level			*p* = 0.498	*p* = 0.608	*p* = 0.556	*p* = 0.334	*p* = 0.718	*p* = 0.003
Junior high school and below	597 (62.38)^a^	2,186 (40.08)^a^	93.13 (91.10–95.17)	97.8 (97.19–98.42)	90.45 (88.09–92.82)	95.24 (94.35–96.14)	83.42 (80.43–86.41)	87.05 (85.65–88.46)
Senior High School	199 (20.79)^a^	1,396 (25.60)^a^	93.97 (90.63–97.31)	98.07 (97.34–98.79)	92.96 (89.38–96.55)	94.27 (93.05–95.49)	81.41 (75.95–86.86)	83.60 (81.65–85.54)
University and above	161 (16.82)^a^	1872 (34.32)^a^	95.65 (92.47–98.84)	98.24 (97.64–98.83)	91.30 (86.91–95.70)	95.30 (94.34–96.26)	84.47 (78.82–90.13)	83.81 (82.14–85.48)
Smoking status			*p* = 0.127	*p* = 0.795	*p* = 0.077	*p* = 0.339	*p* = 0.61	*p* = 0.253
Current smoker	229 (23.93)^a^	1,160 (21.27)^a^	96.51 (94.11–98.9)	98.02 (97.21–98.82)	94.76 (91.85–97.67)	94.22 (92.88–95.57)	88.21 (84.00–92.42)	83.53 (81.4–85.67)
Former smoker	112 (11.72)^a^	287 (5.12)^a^	93.75 (89.20–98.30)	98.57 (97.16–99.97)	91.07 (85.71–96.43)	94.62 (91.96–97.29)	80.36 (72.88–87.83)	84.95 (80.72–89.17)
Never smoker	616 (64.37)^a^	4,015 (73.62)^a^	92.69 (90.63–94.76)	97.98 (97.55–98.42)	89.77 (87.37–92.17)	95.27 (94.61–95.92)	81.82 (78.76–84.87)	85.5 (84.41–86.59)
Drinking status			*p* < 0.001	*p* = 0.457	*p* = 0.002	*p* = 0.800	*p* = 0.001	*p* = 0.682
Current drinker	268 (28.00)^a^	1,394 (25.56)^a^	98.13 (96.5–99.76)	98.42(97.77–99.08)	95.90(93.50–98.29)	94.98(93.83–96.13)	89.93(86.3–93.55)	84.43(82.53–86.34)
Former drinker	93 (9.72)^a^	275 (5.04)^a^	87.10 (80.16–94.04)	97.82 (96.08–99.56)	84.95 (77.54–92.35)	94.18 (91.40–96.97)	76.34 (67.54–85.14)	86.18 (82.08–90.29)
Never drinker	596 (62.28)^a^	3,785 (69.76)^a^	92.79 (90.70–94.87)	97.89 (97.43–98.34)	89.93 (87.51–92.36)	95.09 (94.40–95.77)	81.21 (78.06–84.35)	85.20 (84.07–86.34)
Physical activity			*p* = 0.316	*p* = 0.252	*p* = 0.08	*p* = 0.004	*p* = 0.272	*P* < 0.001
High level	536 (56.00)^a^	2,736 (50.17)^a^	94.78 (92.89–96.67)	98.32 (97.84–98.8)	92.91 (90.73–95.09)	95.98 (95.24–96.72)	4.89 (81.85–87.93)	88.08 (86.87–89.30)
Middle level	257 (26.85)^a^	1769 (32.43)^a^	92.22 (88.92–95.52)	97.63 (96.92–98.34)	88.33 (84.37–92.28)	94.06 (92.96–95.17)	81.32 (76.53–86.12)	83.21 (81.47–84.95)
Low level	200 (17.14)^a^	949 (17.40)^a^	92.68 (88.66–96.71)	97.89 (96.98–98.81)	89.63 (84.92–94.35)	93.99 (92.48–95.51)	80.49 (74.36–86.62)	79.77 (77.21–82.33)
The history of allergic			*p* = 0.227	*p* = 0.179	*p* = 0.014	*p* = 0.304	*p* = 0.030	*p* = 0.051
Yes	112 (11.70)^a^	327 (6.00)^a^	91.07 (85.71–96.43)	96.64 (94.67–98.60)	85.71 (79.13–92.30)	93.27 (90.54–96.00)	75.89 (67.85–83.94)	82.57 (78.44–86.70)
No	740 (77.32)^a^	4,521 (82.73)^a^	94.46 (92.81–96.11)	98.12 (97.72–98.51)	92.57 (90.67–94.46)	95.17 (94.54–95.79)	84.86 (82.28–87.45)	85.59 (84.57–86.62)
Unclear	105 (10.97)^a^	615 (11.28)^a^	91.43 (85.99–96.87)	98.05 (96.95–99.14)	86.67 (80.06–93.28)	94.80 (93.04–96.56)	79.05 (71.13–86.96)	82.44 (79.42–85.45)
Subjective social status in China	5 (3–5)^b^	5 (3–6)^b^	–	–	–	–	–	–
Self-report health condition	72 (60–82)^b^	82 (74–92)^b^	–	–	–	–	–	–
Public health prevention measures			*p* = 0.396	*p* = 0.047	*p* = 0.151	*P* < 0.001	*P* = 0.004	*P* < 0.001
Low level	108 (12.29)^a^	395 (7.24)^a^	90.74 (85.19–96.30)	96.71 (94.94–98.48)	87.04 (80.60–93.47)	91.65 (88.90–94.39)	76.85 (68.77–84.94)	79.49 (75.49–83.49)
Middle level	138 (14.42) ^a^	374 (6.86)^a^	94.20 (90.25–98.15)	97.06 (95.34–98.78)	89.13 (83.87–94.39)	91.44 (88.60–94.29)	76.09 (68.88–83.29)	79.41 (75.29–83.53)
High level	711 (74.29)^a^	4,685 (85.90)^a^	94.09 (92.36–95.83)	98.21 (97.83–98.59)	92.12 (90.14–94.11)	95.58 (94.99–96.17)	85.51 (82.92–88.11)	85.98 (84.98–86.97)
Self-efficacy			*p* < 0.001	*p* < 0.001	*p* < 0.001	*p* < 0.001	*p* < 0.001	*p* < 0.001
Low (4–15)	234 (24.45)^a^	1,102 (20.72)^a^	87.18 (82.86–91.49)	96.28 (95.16–97.40)	82.05 (77.10–87.00)	90.38 (88.64–92.12)	64.53 (58.35–70.71)	72.41 (69.77–75.06)
Moderate (16)	413 (43.16)^a^	2,427 (44.50)^a^	95.16 (93.08–97.24)	98.15 (97.61–98.68)	92.74 (90.22–95.25)	95.63 (94.82–96.45)	86.44 (83.13–89.76)	86.03 (84.65–87.41)
High (17–20)	310 (32.39)^a^	1925 (35.30)^a^	96.77 (94.80–98.75)	98.86 (98.38–99.33)	95.81 (93.56–98.05)	96.88 (96.11–97.66)	92.90 (90.03–95.78)	91.06 (89.79–92.34)

a The number and percentages derived from the total number in the corresponding row.

b The median and interquartile range.

Subjective social status in China: the self-rated social status in the questionnaire increased from 0 to 10.

Self-report health condition: the self-rated health status in the questionnaire increased from 0 to 100.

Among the participants with chronic diseases, 93.70% (95% CI: 92.19–95.27%), 91.12% (95%CI: 94.43–95.59%), and 83.18% (95%CI: 80.80–85.55%) received the first vaccination, the primary vaccination, and the booster vaccination, respectively. At any dose, vaccination rates were higher in participants who are alcohol users than those with and without a history of alcohol consumption.

In the general population, the vaccination rates for the first-dose, second-dose and booster shots were 98.02% (95% CI: 97.65–98.39%), 95.01% (95% CI: 94.43–95.59%) and 85.06% (95% CI: 84.11–86.00%) respectively, which were higher than participants with chronic diseases.

### Vaccination rate of different doses of vaccine among different population

In the first dose of vaccination, vaccination rate remained high across the groups. There were no significant differences in vaccination rates among people with chronic disease, diabetes or hypertension. And vaccination rates in these population were lower than in the general population (*p* < 0.05/6). In contrast, the second dose vaccination rate was found to be lower than that of the first dose, staying below 95 percent. Second dose vaccination rates were higher in the general population than in those with chronic diseases, but there were no significant differences between those with hypertension or diabetes (*p* < 0.05/6). In booster vaccinations, the rate drops to about 85 percent. There was no significant difference between the vaccination rates of the general population and those of patients with chronic diseases (Table 2).

**Table 2:** The result of chi-square test in three types of vaccination among different population.

	General population	Chronic disease population	Hypertension	Diabetes	*p* value
First dose vaccination rate					<0.001
Vaccinated	5,346 (98.11%)^a^	897 (94.30%)^b^	599 (95.66%)^b^	216 (94.78%)^b^	
Unvaccinated	108 (1.89%)^a^	60 (5.70%)^b^	30 (4.34%)^b^	13 (5.22%)^b^	
Second dose vaccination rate					<0.001
Vaccinated	5,182 (93.84%)^a^	872 (89.65%)^b^	581 (91.03%)^b^	210 (89.96%)^b^	
Unvaccinated	272 (6.16%)^a^	85 (10.35%)^b^	48 (8.7%)^b^	19 (10.04%)^b^	
Booster dose vaccination rate					0.166
Vaccinated	4,369 (80.99%)^a^	796 (75.59%)^a^	536 (77.57%)^a^	185 (74.30%)^a^	
Unvaccinated	815 (19.01%)^a^	161 (24.41%)^a^	93 (22.43%)^a^	44 (25.70%)^a^	

^a, b^ The result of the Bonferroni test. ^a^ and ^b^ stand for different groups. There is no significant difference in vaccination rates within the same group.

**Figure 2:**
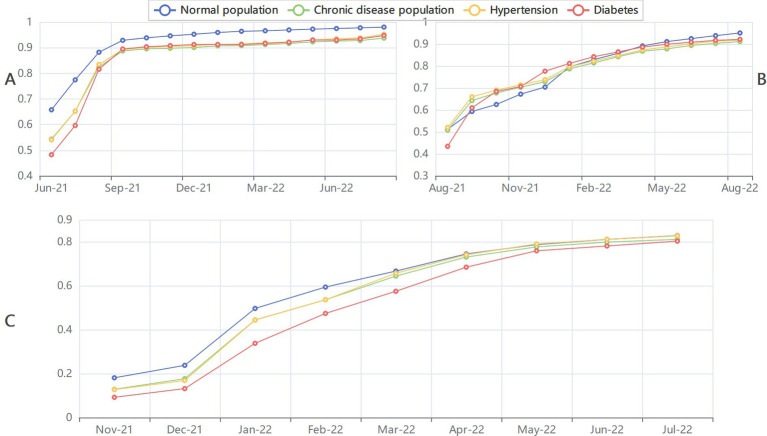
The trend of different dose of vaccination rates among general population chronic disease population hypertension and diabetes. **(A)** The trend of first dose vaccination rates. **(B)** The trend of fully dose vaccination rates. **(C)** The trend of booster dose vaccination rates.

The linear trend of vaccination rate was significant for all doses of the vaccination whereas the vaccination rate increased overtime. At any fixed point in time, the trend of different types vaccination rate was also significant. The vaccination rate decreased with the first dose vaccination, second dose vaccination and booster vaccination. Detailed vaccination rates and the results of all Cochran-Armitage trend tests are presented in Supplementary Table S1. The trend of vaccination rates for different vaccines is shown in Figure 2.

### Factors of the different type vaccination rate

The result of binary logistic regression model is shown in Table 3. The forest plots of factors associated with vaccination are shown in Supplementary Figures S1–S3. Self-efficacy displays the positive effect in the chronic disease and the general population. Across all doses of vaccines, vaccination was associated with greater self-efficacy, and the strength of this association increased as self-efficacy increased (AORs ranged from 1.97 to 6.55, 95% CIs ranged from = 1.28, 3.88 to 3.24, 11.07).

**Table 3:** The influencing factors of vaccine uptake in different stage of COVID-19 vaccination.

Variables	First dose vaccination		Second dose vaccination		Booster dose vaccination	
	Chronic disease population	General population	Chronic disease population	General population	Chronic disease population	General population
	AOR (95%CI)^a^	AOR (95%CI)^b^	AOR (95%CI)^c^	AOR (95%CI)^d^	AOR (95%CI)^e^	AOR (95%CI)^f^
Age, year	1.01 (0.98–1.03)	1.00 (0.98–1.01)	0.99 (0.98–1.01)	1.01 (1.00–1.02)	1.00 (0.98–1.01)	1.01 (1.00–1.02)
Sex						
Male	1.00 (ref.)	1.00 (ref.)	1.00 (ref.)	1.00 (ref.)	1.00 (ref.)	1.00 (ref.)
Female	1.20 (0.65–2.22)	0.81 (0.55–1.20)	1.01 (0.62–1.85)	0.96 (0.75–1.23)	1.22 (0.79–1.88)	1.01 (0.87–1.18)
Educational level						
Junior high school and below	1.00 (ref.)	1.00 (ref.)	1.00 (ref.)	1.00 (ref.)	1.00 (ref.)	1.00 (ref.)
Senior High School	1.29 (0.63–2.63)	1.1 (0.68–1.83)	1.49 (0.78–2.86)	0.94 (0.68–1.28)	0.86 (0.54–1.37)	0.80 (0.65–0.97)
University and above	2.25 (0.87–5.83)	1.33 (0.78–2.26)	1.26 (0.60–2.67)	1.47 (1.05–2.06)	1.11 (0.61–2.00)	0.95 (0.77–1.17)
Social status in China	1.08 (0.95–1.22)	1.02 (0.94–1.11)	1.01 (0.91–1.13)	0.97 (0.92–1.03)	0.99 (0.912–1.08)	0.92 (0.89–0.95)
Smoking status						
Current smoker	1.00 (ref.)	1.00 (ref.)	1.00 (ref.)	1.00 (ref.)	1.00 (ref.)	1.00 (ref.)
Former smoker	1.17 (0.37–3.67)	1.42 (0.48–4.17)	1.04 (0.40–2.72)	0.97 (0.54–1.74)	0.80 (0.40–1.61)	1.02 (0.70–1.48)
Never smoker	0.58 (0.23–1.46)	1.16 (0.64–2.10)	0.61 (0.27–1.36)	1.48 (1.01–2.15)	0.74 (0.40–1.35)	1.25 (0.99–1.57)
Drinking status						
Current drinker	1.00 (ref.)	1.00 (ref.)	1.00 (ref.)	1.00 (ref.)	1.00 (ref.)	1.00 (ref.)
Former drinker	0.14 (0.05–0.40)	0.71 (0.28–1.77)	0.27 (0.12–0.64)	0.77 (0.44–1.36)	0.41 (0.21–0.78)	1.06 (0.82–1.55)
Never drinker	0.22 (0.08–0.59)	0.77 (0.44–1.33)	0.37 (0.17–0.77)	1.04 (0.74–1.45)	0.42 (0.25–0.72)	1.02 (0.83–1.25)
Physical activity						
Low level	1.00 (ref.)	1.00 (ref.)	1.00 (ref.)	1.00 (ref.)	1.00 (ref.)	1.00 (ref.)
Middle level	1.33 (6.45–2.74)	1.05 (0.61–1.81)	0.64 (0.38–1.01)	1.38 (0.99–1.92)	1.18 (0.73–1.93)	1.56 (1.27–1.91)
High level	0.94 (0.44–2.02)	0.80 (0.46–1.40)	0.72 (0.39–1.35)	0.99 (0.70–1.38)	1.06 (0.63–1.81)	1.17 (0.95–1.44)
The history of allergic						
Yes	1.00 (ref.)	1.00 (ref.)	1.00 (ref.)	1.00 (ref.)	1.00 (ref.)	1.00 (ref.)
No	1.34 (0.63–2.86)	1.69 (0.88–3.23)	2.64 (1.59–4.39)	1.19 (0.75–1.89)	1.45 (0.86–2.49)	1.05 (0.77–1.43)
Unclear	0.93 (0.35–2.49)	1.92 (0.83–4.44)	4.52 (2.34–8.73)	1.35 (0.77–2.39)	1.15 (0.58–2.30)	1.02 (0.71–1.47)
Self-report health condition	1.12 (1.00–1.03)	1.00 (0.99–1.01)	1.01 (1.00–1.03)	1.01 (1.00–1.02)	1.01 (1.01–1.03)	1.00 (1.00–1.01)
Public health prevention measures						
Low level	1.00 (ref.)	1.00 (ref.)	1.00 (ref.)	1.00 (ref.)	1.00 (ref.)	1.00 (ref.)
Middle level	1.70 (0.63–4.61)	1.15 (0.51–2.61)	1.23 (0.55–2.75)	1.01 (0.60–1.70)	0.91 (0.49–1.72)	1.02 (0.72–1.47)
High level	1.30 (0.61–2.78)	1.55 (0.85–2.84)	1.26 (0.66–2.50)	1.67 (1.13–2.50)	1.29 (0.76–2.20)	1.29 (0.98–1.68)
Self-efficacy						
Low (4-15)	1.00 (ref.)	1.00 (ref.)	1.00 (ref.)	1.00 (ref.)	1.00 (ref.)	1.00 (ref.)
Moderate (16)	2.89 (1.59–5.25)	1.97 1.28–3.24)	2.64 (1.59–4.39)	2.15 (1.62–2.85)	3.38 (2.27–5.03)	2.25 (1.88–2.69)
High (17-20)	4.30 (2.04–9.08)	3.14 (1.85–5.35)	4.52 (2.34–8.73)	2.89 (2.06–4.03)	6.55 (3.88–11.07)	3.51 (2.85–4.33)

a Adjusted age, gender, drinking status, and self-efficacy.

b Adjusted age, gender, and self-efficacy.

c Adjusted age, gender, drinking status the history of allergic, and self-efficacy.

d Adjusted age, gender, physical activity, public prevention measures, and self-efficacy.

e Adjusted age, gender, drinking status, physical activity, the history of allergic, public prevention measures, and self-efficacy.

f Adjusted age, gender, educational level, physical activity, the history of allergic, public prevention measures, and self-efficacy.

Higher education level (AOR = 1.47, 95%CI: 1.05, 2.06), middle level physical activity (AOR = 1.56, 95%CI: 1.27–1.91) and higher public prevention measures (AOR = 1.67, 95%CI: 1.13–2.50) play a positive role in vaccination among the general population. Compared to the general population, alcohol consumption acts as a significant factor in the chronic disease population. In any dose of the vaccine, participants with no history of alcohol consumed (AORs range from 0.14 to 0.42, 95%Cis ranged from 0.05, 0.25 to 0.42, 0.72) had a lower probability of being vaccinated compared to participants who were currently drinking.

### The reason for not getting the booster vaccinated

**Figure 3:**
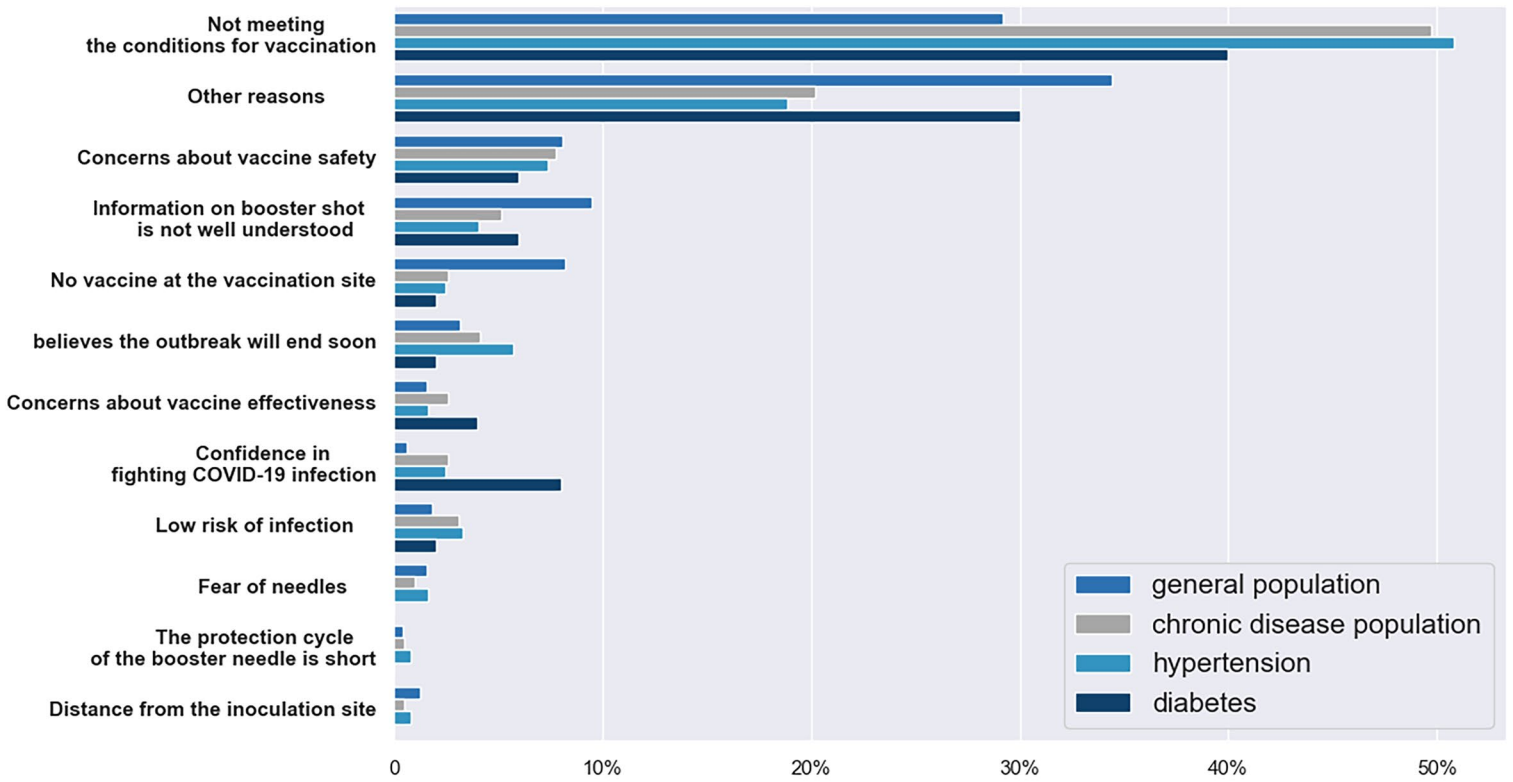
The reason of participants not getting the booster vaccine. (This figure shows the percentage of reason why participants did not get the booster vaccine. In some groups, no participant chose the options, so the option only has three bars).

The Figure 3 depicts reasons for participants not to receive the booster vaccine. In chronic disease populations, not meeting vaccination requirements was the primary barrier to booster vaccination, followed by other factors, such as concern about vaccine safety and a lack of understanding about booster shots whereas other reasons account for the largest proportion in general population.

## Discussion

We analyzed the trend of vaccination rates among chronic disease and the general population. It was found that the vaccination rate in three doses was in a stagnant state of growth and the vaccination rate showed a downward trend with the increase in the number of vaccinations. Our study divided the population into two categories to study vaccination, which has important theoretical implications for revealing the development pattern of vaccination rate and further improving vaccination rate.

Currently, vaccination rates have been stagnant. In the chronic diseases population, the first dose vaccination rate rose rapidly until September 2021, after which it maintained a slight increase and reached 93.73% in August 2022. The same situation happened in second dose vaccination rate and the booster vaccination rate, reaching 91.12 and 83.18%, respectively. Currently, vaccination rates for each dose showed a gradual increase. Without the intervention, vaccination rates would have remained at this level and hardly increased significantly. We also can find an obvious downward trend in vaccination rates from first dose to booster dose. This phenomenon reflects that a shift in public attitudes toward the pandemic and the vaccine. Studies have shown that vaccine hesitancy increases significantly during the pandemic (18, 19). Similar to the conclusion of previous studies, the declining trend of vaccination willingness was also reflected in the vaccination. Although most people receive the booster shots to strengthen the immunity, the vaccine effectiveness would wane overtime and decline because of virus variants such as omicron (7, 20, 21), and this decline was even more pronounced among elder people with chronic diseases such hypertension and diabetes (22).

As China relaxes epidemic control measures, this will accelerate the speed of the spread of the COVID-19 and the likelihood of people becoming infected. Due to China’s large population, this could lead to a long-term shortage of medical resources and difficult access to medical services. Flattening the curve is one way to ease the pressure on the health system. Regular vaccination is an effective way to curb the spread of the virus under the relaxed policy. In China the fourth dose of the vaccine has only been rolled out on a small scale. If this downward trend persists, the next round of vaccinations will have a lower coverage rate and a larger gap compared to the booster vaccine. Insufficient vaccine coverage will lead to more frequent outbreaks (23), which increases the economic burden and waste of public health resources. It is essential for public policymaker to be aware of this declining vaccination trend in case future vaccines fail to achieve the desired effect.

Alcohol consumption uniquely influence vaccination among chronic disease population. Alcohol, a risk factor associated with kinds of infection, suppresses individual’s immune response resulting in higher risk of contracting the virus (24, 25). Similarly, alcohol consumption increases the neutrophil and neutrophil to lymphocyte ratio and reduce the number of NK cells, T cells, B cells which will cause a more serious outcomes after infection with COVID-19 (26). Alcohol consumption is associated with gathering activities, which also increases the odds of infection (27). Although one study found no association between alcohol consumption and the probability of infection and severity of infection (28), most studies still indicate that there is an association between the two (29, 30). Chronic diseases status and the factors mentioned may make such people treat epidemic with more caution and thus lead to higher vaccination rate. However, a large cross-sectional study in China showed that people who never or rarely drank alcohol had a lower likelihood of vaccine hesitancy than those who drank alcohol frequently (31). We think there are two possible explanations for this difference. First, the two studies were conducted at different times, and the perception of vaccination may have changed as the epidemic progressed. Second, because of the difference in the population analyzed, our finding only applies to the chronic disease group, while the previous finding only applies to all participants, and this difference is what we want to get by dividing the population into two groups for analysis.

Higher self-efficacy was a significant factor in promoting vaccination, which has been observed in all doses of vaccines, in people with chronic diseases and in the general population. A previous study which applied an extended protection motivation theory to predict covid-19 vaccination intentions and uptakes found that higher level of self-efficacy is related to both vaccination intentions and uptake (32), which is consistent with our result. There are more studies showing the link between self-efficacy and vaccination intention (33, 34). In addition, self-efficacy is not just related to vaccination intention and behavior. It is also related to the adherence to covid-19 preventive measures and the self-isolation intention during the period of epidemic (35, 36), which is important for reducing the number of infections because even vaccination requires social distancing and protective measures (37). This shows that self-efficacy has played a crucial role in the covid-19 pandemic. How to improve self-efficacy is a still a question worth discussing. Overloading information have a negative effect on self-efficacy, which can be explained that mass information obscures the real information (36). A cross-section study in China found that higher perceived benefits and lower perceived risks has the positive impact on self-efficacy (38). The other study reveals that vaccine safety and vaccine side effects are related to the self-efficacy (39), whereas another study emphasized the importance of trust in traditional media in promoting self-efficacy (40). According to our result and aforementioned studies, policymakers should pay attention to the role of self-efficacy in epidemic prevention. Effective methods for enhancing self-efficacy include disseminating accurate information through authoritative media and controlling the Network environment to reduce redundant vaccine information. The public should be informed about the latest vaccine policy for people with chronic diseases, the effectiveness of vaccination, the safety of vaccine and the side effects that are not worth worrying about.

The main reason for the population with chronic diseases who did not receive booster vaccines is not meeting the condition of vaccination. The joint prevention and control mechanism of The State Council has made corresponding answers to the question of whether people with chronic disease can be vaccinated against COVID-19. Patients with chronic diseases, including hypertension, diabetes, cancer and COPD, should not be considered as taboo for covid-19 vaccination as long as their health conditions is stable and under drug control. During the pandemic, changes in lifestyle may cause more chronic patients to be in an unstable state and thus not meet the condition of vaccination (41, 42). Some people who choose this option may also mistakenly believe that chronic diseases are unsuitable for vaccination. Advocating a healthy lifestyle during the epidemic, more publicity about the safety of vaccines, and letting more people know whether vaccinations are available for chronic disease are effective ways to address concerns about booster vaccination in people with chronic diseases.

For now, vaccination rates for both primary and booster shots are at a high level in China, and it is not easy to further increase vaccination rates. Widespread awareness and mobilization, as in the early stages of the epidemic, may have little effect and may not be cost-effective. Targeted actions for populations with low vaccination rates may be an effective way to increase vaccination rates further. In addition, we need to be alert to the fact that the next round of vaccination coverage may be low, as more and more infections maybe hinder vaccination.

We recognize several limitations to our study. First, although we noted several factors that appeared to be associated with vaccination, the cause-and-effect relationship can not be inferred from our research. Additionally, we determined whether the participants had chronic diseases based on the questionnaires self-administered by the participants, and did not rely on data from medical institutions. It is possible that some participants with chronic diseases did not want to disclose their information and chose to conceal their disease history, which may have leaded to information bias. Finally, this survey is household-based and its results may not be applicable to the mobile population. Nevertheless, our study has many strengths. Our study examined vaccination rates by dose and trends in the chronic disease population, adding to the limited information available on vaccination in the chronic disease population. In addition, we further investigated vaccination, which better reflects the factors influencing vaccination rates compared to vaccination intentions. We also divided the participants into two groups by the presence or absence of chronic diseases, which contributed to the discovery of some hidden information. Finally, this study is only a baseline study of the vaccination cohort, and the subsequent three phases of follow-up are in progress in an orderly manner.

## Conclusion

The primary vaccination rate for COVID-19 in the Chinese population has been at a high level. Although the rate of booster vaccination is also at a relatively high level, there is still room for increase the rate of booster vaccination compared to the primary vaccination rate. In addition, as the number of vaccinations increases, the trend of decreasing vaccination rate is becoming more pronounced. In future regular vaccinations, we may face low vaccination rates as the increasing number of infections and the fatigue associated with the prolonged outbreak hamper vaccination. Measures need to be found to counter this downward trend such as improving the self-efficacy of the population.

## Data availability statement

The raw data supporting the conclusions of this article will be made available by the authors, without undue reservation.

## Ethics statement

The studies involving human participants were reviewed and approved by Life Science Ethics Review Committee of Zhengzhou University (Approval number: 2021-01-12-05). The patients/participants provided their written informed consent to participate in this study.

## Author contributions

YM, JW, XG, MW, JG, and BY: conceptualization. YM, XG, and YH: data curation. JW, XG, YM, and XZ: formal analysis. MW and JG: funding acquisition. JW, YM, XG, YX, CT, and XZ: investigation. YM, JW, XG, and CT: methodology and writing – original draft. JW: project administration. JW and YM: resources. XG, YX, and BY: software. YM, CT, MW, JG, and BY: writing – review and editing. All authors contributed to the article and approved the submitted version.

## Funding

This work was supported by Henan Provincial Philosophy and Social Science Innovation Talent Support Plan; Grant number: 2023-CXRC-06. Platform for Dynamic Monitoring and Comprehensive Evaluation of Healthy Central Plains Action; Grant number: 20220134B. 2021 Postgraduate Education Reform and Quality Improvement Project of Henan Province; Grant number: YJS2021KC07. 2021 Graduate Independent Innovation Project of Zhengzhou University Grant number: 20211207.

## Conflict of interest

The authors declare that the research was conducted in the absence of any commercial or financial relationships that could be construed as a potential conflict of interest.

## Publisher’s note

All claims expressed in this article are solely those of the authors and do not necessarily represent those of their affiliated organizations, or those of the publisher, the editors and the reviewers. Any product that may be evaluated in this article, or claim that may be made by its manufacturer, is not guaranteed or endorsed by the publisher.
