# Supplementary material for: The pattern from the first three rounds of vaccination: declining vaccination rates
Source: Front Public Health. 2023 May 12;11:1124548. doi: 10.3389/fpubh.2023.1124548 (PMC10213674; doi:10.3389/fpubh.2023.1124548)
Supplement: Supplementary file 1 [file Data_Sheet_1.docx]

**Supplementary legends**

**Supplementary Table 1** The Trend of first dose of vaccination rate, fully vaccination rate and booster vaccination rate in different population.

**Supplementary Figure 1** Factors associated with first dose vaccination.

**Supplementary Figure 2** Factors associated with second dose vaccination.

**Supplementary Figure 3** Factors associated with booster vaccination.

|  | Nov  21 | Dec  21 | Jan  22 | Feb  22 | Mar  22 | Apr  22 | May  22 | Jun  22 | Jul  22 | Aug  22 |  |
| --- | --- | --- | --- | --- | --- | --- | --- | --- | --- | --- | --- |
|  |  |  |  |  |  |  |  |  |  |  | *P* for trend ^b^ |
| **Normal population** |  |  |  |  |  |  |  |  |  |  |  |
| First dose of vaccination | 94.61% | 95.23% | 95.93% | 96.39% | 96.59% | 96.92% | 97.23% | 97.47% | 97.74% | 98.02% | <0.001 |
| Fully Vaccinated | 67.18% | 70.39% | 79.57% | 82.89% | 85.77% | 89.15% | 91.09% | 92.45% | 93.80% | 95.01% | <0.001 |
| Booster vaccinated | 18.08% | 23.74% | 49.65% | 59.37% | 66.65% | 74.44% | 78.71% | 81.08% | 82.78% | 85.06% | <0.001 |
| *P* for trend ^a^ | <0.001 | <0.001 | <0.001 | <0.001 | <0.001 | <0.001 | <0.001 | <0.001 | <0.001 | <0.001 |  |
| **Chronic disease population** |  |  |  |  |  |  |  |  |  |  |  |
| First dose of vaccination | 89.76% | 90.07% | 90.70% | 90.80% | 91.22% | 91.64% | 92.27% | 92.58% | 92.79% | 93.73% | 0.04 |
| Fully Vaccinated | 70.32% | 72.83% | 78.68% | 81.40% | 84.22% | 86.73% | 87.77% | 89.34% | 90.28% | 91.12% | <0.001 |
| Booster vaccinated | 12.85% | 17.66% | 44.41% | 53.61% | 64.37% | 73.04% | 77.64% | 79.83% | 81.09% | 83.18% | <0.001 |
| *P* for trend | <0.001 | <0.001 | <0.001 | <0.001 | <0.001 | <0.001 | <0.001 | <0.001 | <0.001 | <0.001 |  |
| **Hypertension** |  |  |  |  |  |  |  |  |  |  |  |
| First dose of vaccination | 90.46% | 90.78% | 91.26% | 91.41% | 91.89% | 92.21% | 93.00% | 93.48% | 93.80% | 94.91% | 0.006 |
| Fully Vaccinated | 71.38% | 73.77% | 79.65% | 82.19% | 84.74% | 87.28% | 88.71% | 90.14% | 91.26% | 91.73% | <0.001 |
| Booster vaccinated | 12.72% | 16.85% | 44.36% | 53.58% | 65.66% | 74.09% | 79.01% | 81.08% | 82.67% | 83.78% | <0.001 |
| *P* for trend | <0.001 | <0.001 | <0.001 | <0.001 | <0.001 | <0.001 | <0.001 | <0.001 | <0.001 | <0.001 |  |
| **Diabetes** |  |  |  |  |  |  |  |  |  |  |  |
| First dose of vaccination | 90.79% | 91.23% | 91.23% | 91.23% | 91.67% | 92.11% | 92.98% | 92.98% | 93.42% | 94.30% | 0.014 |
| Fully Vaccinated | 70.61% | 77.63% | 81.14% | 84.21% | 86.40% | 88.60% | 89.91% | 90.79% | 91.67% | 91.67% | <0.001 |
| Booster vaccinated | 9.21% | 13.16% | 33.77% | 47.37% | 57.46% | 68.42% | 75.88% | 78.07% | 80.26% | 80.70% | <0.001 |
| *P* for trend | <0.001 | <0.001 | <0.001 | <0.001 | <0.001 | <0.001 | <0.001 | <0.001 | <0.001 | <0.001 |  |

**Supplementary Table 1.** The Trend of first dose of vaccination r ate, fully vaccination rate and booster vaccination rate in different population

*P* for trend ^a^: At a fixed time, the *P* value of the trend of first dose of vaccination rate, fully vaccination rate and booster vaccination rate.

*P* for trend ^b^: At a fixed vaccination, the *P* value of the trend of the vaccination rate with the time.


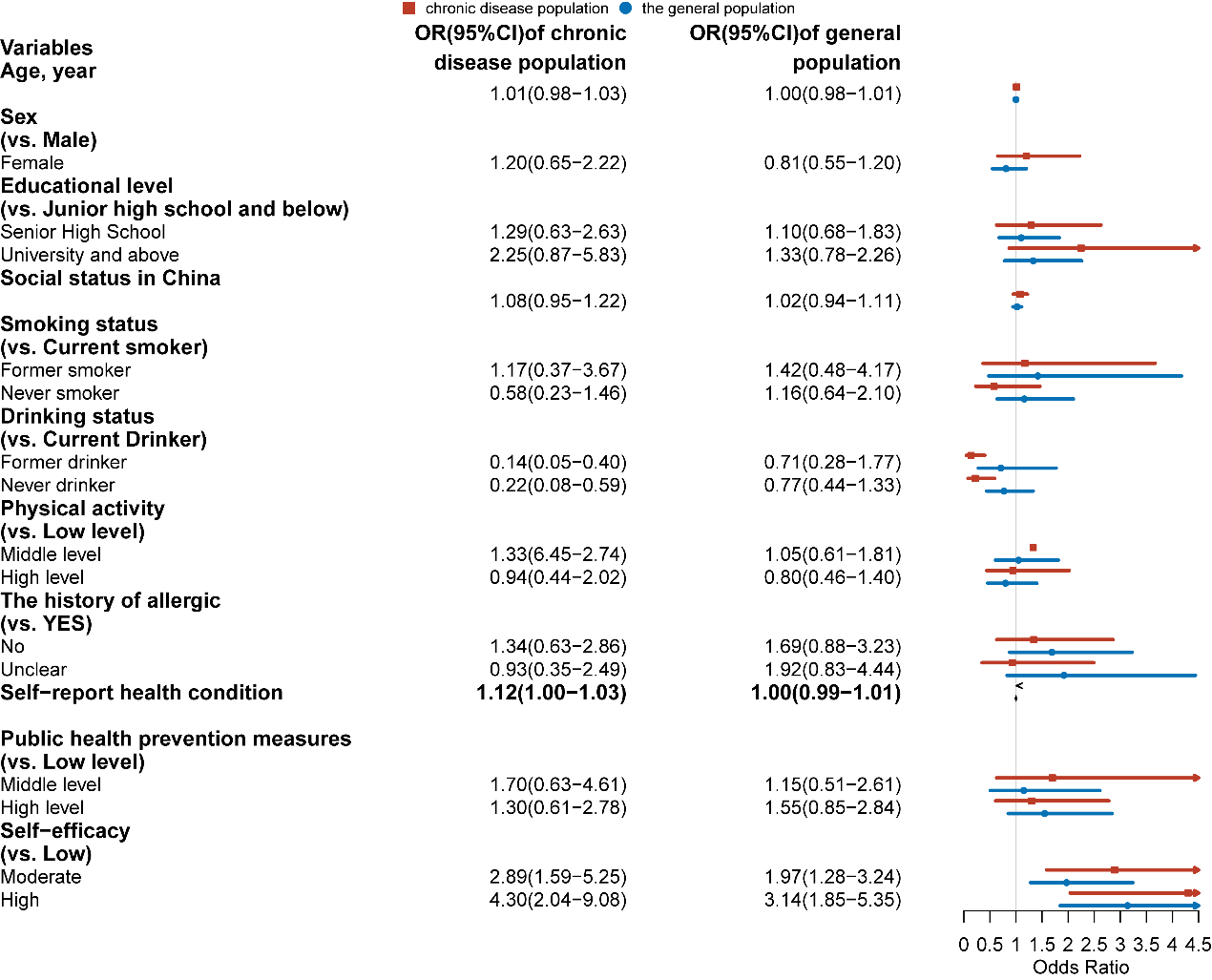


**Supplementary Figure 1.** Factors associated with first dose vaccination.


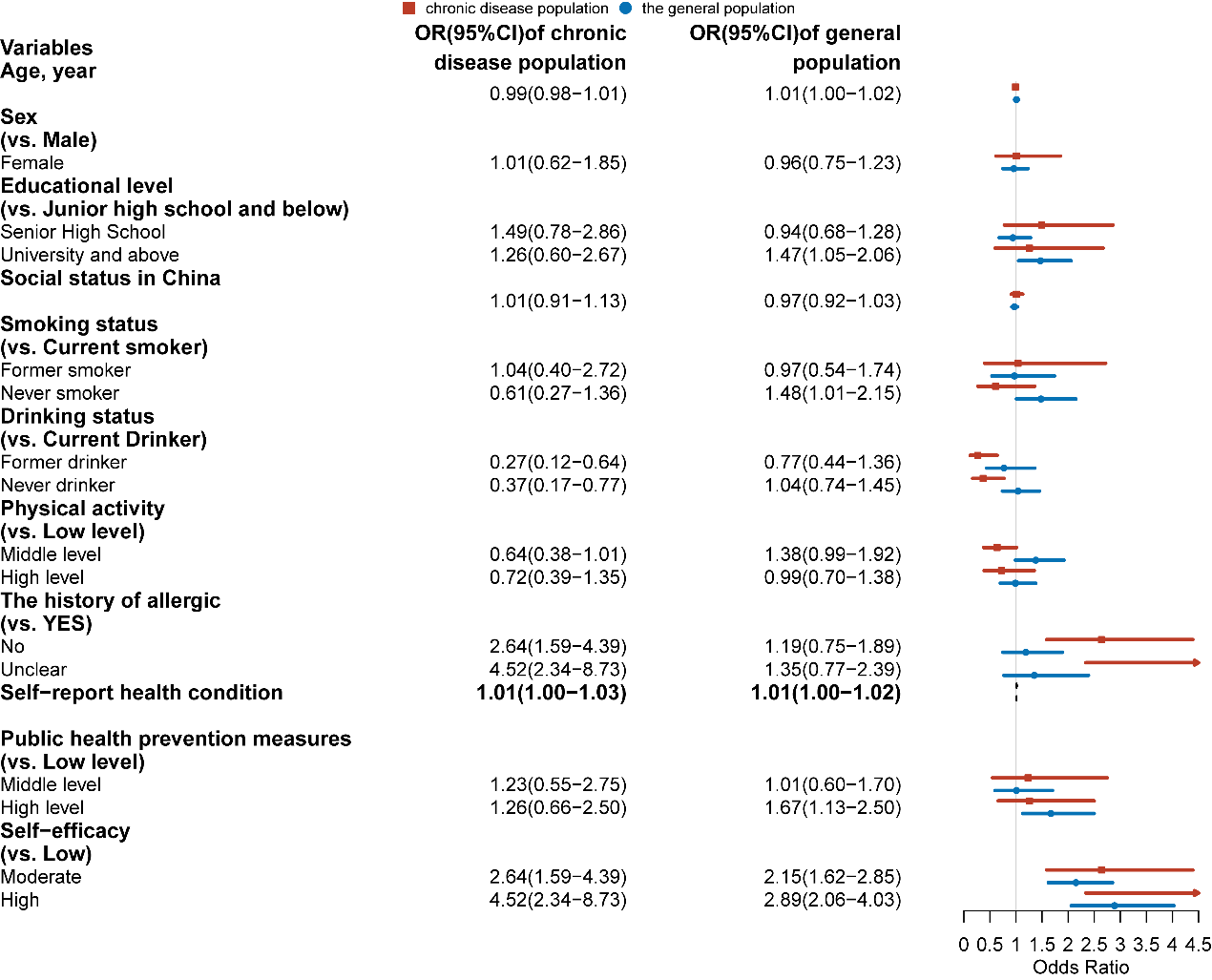


**Supplementary Figure 2.** Factors associated with second dose vaccination.


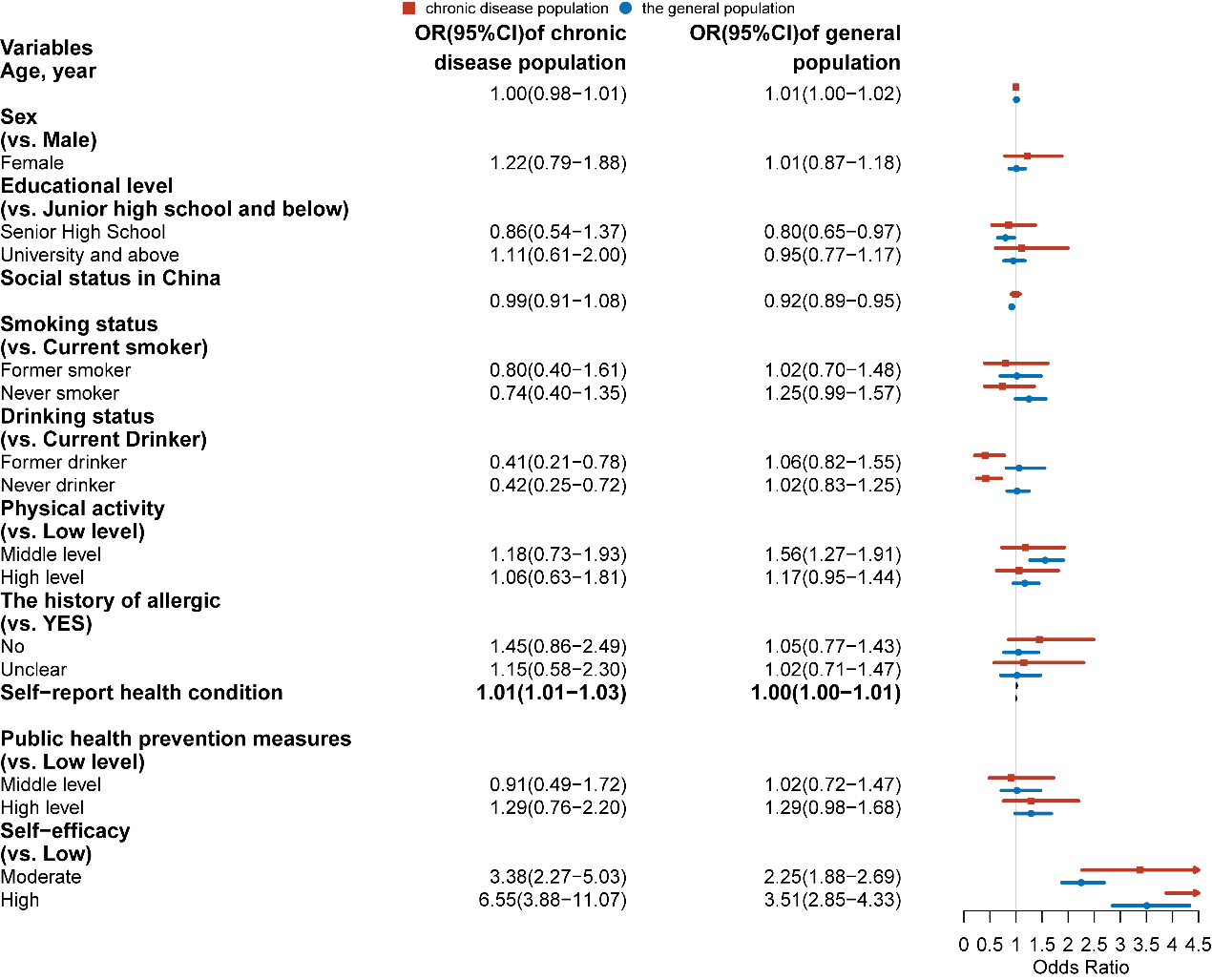


**Supplementary Figure 3.** Factors associated with booster vaccination.
